# Reinforcement Learning-Based Control for Collaborative Robotic Brain Retraction

**DOI:** 10.3390/s24248150

**Published:** 2024-12-20

**Authors:** Ibai Inziarte-Hidalgo, Estela Nieto, Diego Roldan, Gorka Sorrosal, Jesus Perez-Llano, Ekaitz Zulueta

**Affiliations:** 1 Research & Development Department, Aldakin, 31800 Altsasu, Spain; 2 Ikerlan Technology Research Centre, Basque Research and Technology Alliance (BRTA), 20500 Arrasate-Mondragon, Spain; enieto@ikerlan.es (E.N.); droldan@ikerlan.es (D.R.); gsorrosal@ikerlan.es (G.S.); 3 Tedcas Medical Systems, 31013 Pamplona, Spain; jpl@tedcas.com; 4 Faculty of Engineering in Bilbao, University of the Basque Country (UPV/EHU), 48013 Bilbao, Spain; ekaitz.zulueta@ehu.eus

**Keywords:** brain retraction, ROS, reinforcement learning control

## Abstract

In recent years, the application of AI has expanded rapidly across various fields. However, it has faced challenges in establishing a foothold in medicine, particularly in invasive medical procedures. Medical algorithms and devices must meet strict regulatory standards before they can be approved for use on humans. Additionally, medical robots are often custom-built, leading to high costs. This paper introduces a cost-effective brain retraction robot designed to perform brain retraction procedures. The robot is trained, specifically the Deep Deterministic Policy Gradient (DDPG) algorithm, using reinforcement learning techniques with a brain contact model, offering a more affordable solution for such delicate tasks.

## 1. Introduction

In recent years, artificial intelligence (AI) has evolved significantly across various fields, and its impact on healthcare and medicine is becoming increasingly profound [1,2]. AI technologies, including machine learning, deep learning, and natural language processing, are being applied to a wide range of medical tasks such as diagnostics, treatment planning, drug development and personalized medicine. These technologies enable healthcare providers to analyze vast amounts of data quickly and accurately, improving decision making and patient outcomes. AI is also helping to identify patterns and insights that were previously difficult to detect, making it a powerful tool in advancing medical research and improving the overall quality of healthcare delivery.

Although AI is already widely used in medicine, the strict regulatory requirements for algorithms, devices, and robots used in medical applications make innovation in invasive procedures particularly challenging [3]. As a result, most advancements in medical AI have been focused on non-invasive tasks such as diagnosis, drug development and health monitoring [1,2]. The surgical field, in particular, faces significant barriers, as new technologies not only need to meet stringent approval standards but also require expensive, specialized hardware (e.g., the Da Vinci surgical robot).

**Figure 1:**
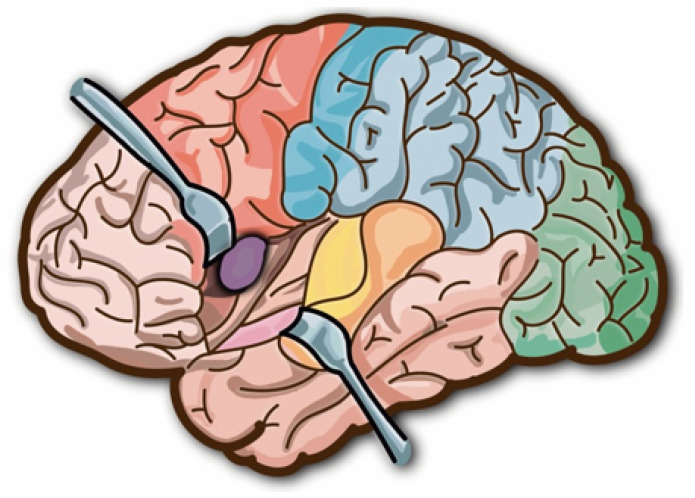
Brain retraction.

This paper focuses on neurological surgeries, specifically brain retractions. Neurological surgery is a specialty that deals with the treatment of diseases affecting the central and peripheral nervous systems. In certain intracranial procedures, surgeons must perform a maneuver called brain retraction, where part of the brain is temporarily moved aside to access deeper regions for treatment (see Figure 1).

While brain retraction is essential for proper tissue exposure during surgery, the incidence of brain injury or damage is not uncommon and varies depending on the definition or consideration of brain damage and can be as high as 47% if edema is considered. According to the literature, the pressure exerted on the brain surface during retraction can cause various types of damage, depending on factors such as the magnitude of the retraction, its duration, and the site of application [4]. This pressure is transmitted to the surrounding brain tissue, leading to deformation and the partial or total closure of blood vessels, which disrupts the oxygen supply to brain cells.

A 2019 study [5] estimates that 22.6 million patients suffer neurological injuries each year, with 13.8 million requiring surgery. Ischemic injuries caused by retraction can lead to cerebral infarction, resulting in contusions, edema, and local tissue necrosis. Deep lesions and procedures are particularly prone to ischemic damage, as greater retraction is often required to access these areas. The neurological symptoms of a surgical stroke resemble those of other strokes, ranging from an asymmetrical smile and limb weakness to speech disorders and, in severe cases, death. Hemorrhagic infarction is another serious risk, arising from vessel occlusion at points of high retractor pressure or as a repercussion after retractor removal [6].

The severity of the injuries mentioned depends on several factors, including the distribution of retractor pressure, the geometry and physical properties of brain tissues, vascular pressure, and the duration of retraction [4]. As a result, neurosurgeons require extensive experience to master the technique of retraction. Even so, they are not immune to issues such as tremors or involuntary movements during prolonged procedures. In contrast, robots can perform repetitive, long-duration tasks without compromising accuracy or stability. However, traditional robotic position controllers have limited adaptability [7], which presents a challenge in automating this medical procedure. Sudden changes in brain pressure distribution can occur, potentially causing damage if the retraction position is not corrected in time.

AI-based controllers, used in industrial applications, have demonstrated their ability to adapt to unknown situations or highly dynamic environments [8]. However, no solutions have been proposed in the literature for critical operations such as brain retraction. In this paper, we present an adaptive and safe controller for robotic brain retraction. The robot autonomously performs a retraction using a DDPG algorithm-based adaptive impedance controller. The robot learns how to execute the retraction through a qualitative simulator that models the contact forces during the procedure, which is later deployed in a real environment. The proposed controller is capable of adjusting its control response to various scenarios and brain characteristics without compromising safety. Additionally, this solution is not dependent on any specific hardware and is compatible with multiple collaborative robots, enhancing the cost-effectiveness of surgical robotics.

The remainder of this paper is organized as follows: Section 2 describes the simulated and real environments used during the controller design process, as well as the ROS-based control architecture proposed for both. Section 3 introduces a novel reinforcement learning-based controller design for brain retraction. The results obtained from both simulations and real-world environments are presented in Section 4. Finally, the discussion and conclusions are presented in Section 5 and Section 6, respectively.

## 2. Materials and Methods

The AI-based controller was designed and parameterized using an iterative methodology. This process was carried out within a software-in-the-loop architecture, which enabled the initial design and tuning of the controller in a simulated environment. The simulation emulates the contact forces between the tool and the brain during retraction procedures. Through an iterative approach, a reinforcement learning algorithm was designed and refined to enhance the controller’s adaptability to various brain behaviors during the retraction process. Specifically, the Deep Deterministic Policy Gradient (DDPG) algorithm was selected to address this particular challenge.

After the control algorithm was developed and pre-trained, it was deployed in a real environment for further tuning and validation. In this setting, the retraction procedure was executed using a robotic arm, and the different behaviors and stiffness levels of various brain tissues were simulated through a pneumatic circuit with adjustable pressure.

The simulation environment was built using Simulink (R2021a)^®^, and the details of the real-world application environment, along with the proposed control architecture, are presented in the following sections.

### 2.1. Simulation Environment

The core of the simulation environment is the contact model, which is based on an impedance system consisting of two components: a spring–damper system representing the environment (brain) and a spring–damper–mass system representing the robot. The impedance system is defined as follows:

$$K_{r}\Delta x_{r}+D_{r}\Delta {\dot{x}}_{r}+M_{r}\Delta {\ddot{x}}_{r}=F=K_{e}\Delta x_{e}+D_{e}\Delta {\dot{x}}_{e}$$

where $K_{r}$ and $K_{e}$ represent the stiffness of the robot and the environment (brain), respectively, $D_{r}$ and $D_{e}$ denote the damping properties of the robot and the environment (damper), $M_{r}$ refers to the mass of the robot, $x_{r}$ is the position of the robot’s TCP (tool center point), $x_{e}$ is the position of the contact point between the environment and the robot, and *F* is the force measured at the contact point. The input of the contact model is $x_{r}$, and the outputs are $x_{e}$ and *F*.

**Figure 2:**
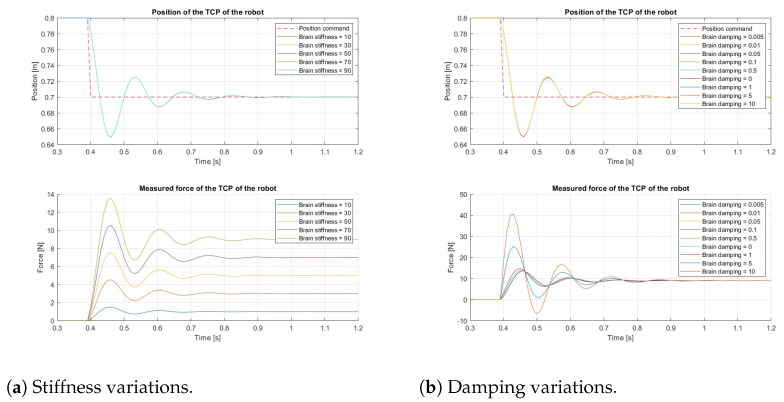
Contact model retraction example.

Figure 2 illustrates the effects of varying the brain impedance on the overall contact model, with each signal representing a different variation. In Figure 2a, the contact position and force of the robot are shown when the stiffness of the brain is adjusted. Figure 2b, on the other hand, displays the contact position and force when the brain damping is altered. The input to the contact model is the position command from the control system, depicted as the intermittent red line in both position graphs in Figure 2. In both cases, the command is a 0.1 m step input. The output of the contact model is the resulting contact position and force. Since the robot is significantly stiffer than the brain, the contact position remains unchanged from one retraction to the next, even when the brain’s impedance varies, as shown in the position graphs. However, the contact force changes with variations in the impedance of the brain. As illustrated in Figure 2a, an increase in brain stiffness results in a higher contact force. If the stiffness continued to increase, the contact position would eventually begin to change. Regarding the damping variation (Figure 2b), the overshoot of the contact force is affected, rather than its steady-state value. These resulting contact forces are generated for a single point of contact since the dispersion of these forces on the contact surface was considered not to be significant for the retraction operation.

### 2.2. Real Environment

**Figure 3:**
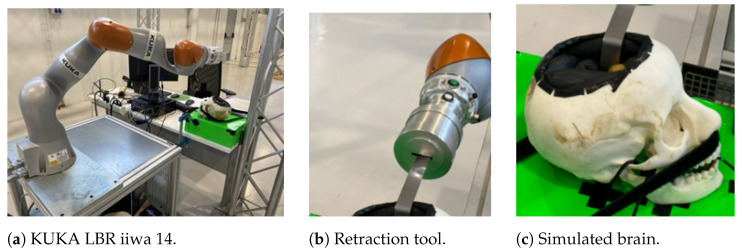
Real test environment.

The real environment, similar to the simulation environment, consists of a robot and a pneumatic system that simulate the physical stiffness and damping characteristics of the brain. By adjusting the pressure, it is possible to simulate different variable stiffness and damping behaviors. The robot used is a KUKA LBR iiwa (KUKA AG., Augsburg, Germany) collaborative robot (see Figure 3a), which was equipped with a retractor (see Figure 3b). Figure 3c displays the pneumatic brain model inserted into an osteopathic skull.

### 2.3. ROS-Based Control Architecture

**Figure 4:**
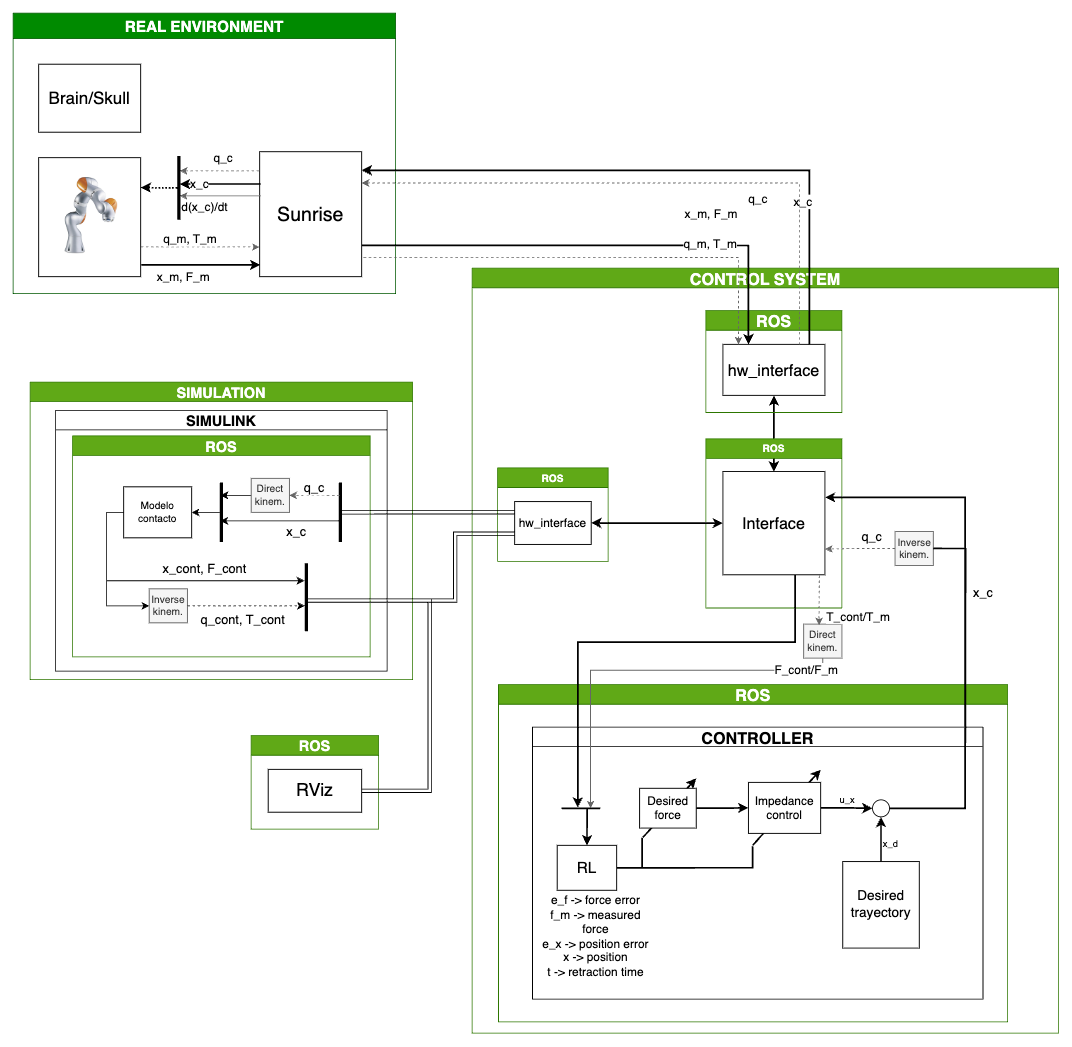
Simulation architecture.

As mentioned above, the simulation environment is critical not only for developing the control system but also for training and later implementing the DDPG algorithm in a real environment. For this reason, the control system’s architecture was designed to facilitate seamless switching between the simulation and real environments. This switching mechanism is illustrated in Figure 4. The simulation environment is represented in the green box, the real environment in the blue box, and the control system in the orange box. The smooth transition between environments is made possible by the hardware interface network, as shown in the figure. Both environments have a custom interface that converts their inputs into a predefined structure. The common interface then processes these inputs, sending them to the controller and the DDPG algorithm, and similarly manages the outputs.

The interface of the real environment of the robot, was already developed and published by [9]. This interface, named iiwa_stack, allows the communication with the KUKA Sunrise through ROS topics, controlling the LBR Iiwa robot through the interface Servoing. So, taking into account that the iiwa_stack interface was already available, the simulation interface was developed to resemble the existing interface of the robot.

The communication between the LBR Iiwa robot and the control system is handled via the topics “sim/tcp_pose” and “sim/tcp_force”, published by the LBR Iiwa robot, and “controller/tcp_command”, which is published by the control system. The control system subscribes to the first two topics, which are “geometry_msgs::Pose”- and “geometry_msgs::Point”-type messages, respectively. The “sim/tcp_pose” topic provides the position and quaternion orientation of the robot’s TCP, while the “sim/tcp_force” topic provides the measured force. The LBR Iiwa robot subscribes to the “controller/tcp_command” topic, which contains the position command generated by the impedance controller. When the robot is not performing a retraction, this command corresponds to the home position; during retraction, it is computed based on the impedance equation and the DDPG algorithm’s output (which adjusts the stiffness, damping, and desired force of the controller).

In addition, to better replicate a real environment, the contact model and the control system were run on separate computers. The control system was deployed on a machine running Ubuntu 20 with ROS Melodic, with the DDPG algorithm and the controller functioning as separate nodes. To prevent desynchronization between the environment and the control system, all components of the system must be synchronized to the same clock. In the real environment, all control system nodes, including the robot, are synchronized to the ROS master clock. However, when using the contact model in a simulation, the model’s computational load requires that the clock be set by the contact model itself.

## 3. Reinforcement Learning Control

The DDPG algorithm has gained increasing importance in robotic control in recent years. It enables a robot to autonomously discover optimal behaviors through trial-and-error interactions with its environment. The key distinction between reinforcement learning and by extension of the DDPG algorithm and other learning approaches is that, instead of providing a detailed solution to a problem, feedback is given in the form of a scalar function, known as a reward, which evaluates the appropriateness of the action taken by the agent as it transitions to a new state [10,11]. Through repeated interactions with the environment, an DDPG algorithm can independently learn the optimal behavior of the system, simplifying operational complexity and ultimately achieving greater control than traditional intelligent algorithms [12]. In this study, the control system for the LBR iiwa retraction robot is designed as a reinforcement learning-based adaptive impedance controller. In other words, the LBR iiwa robot is controlled by an impedance controller, which is continuously adapted by an RL algorithm. The DDPG algorithm’s action is defined as

$$a=[K_{c},D_{c},F_{SP}]$$

where *a* represents the action, $K_{c}$ and $D_{c}$ the stiffness and damping of the controller, respectively, and $F_{SP}$ is the desired (or set point) force. The state is defined as

$$s=[F_{m},{\dot{F}}_{m},K_{c},D_{c},e_{x},\dot{x}]$$

where *s* represents the state, $F_{m}$ is the measured force, $e_{x}$ is the position error of the robot’s TCP, and *x* is the position of the robot’s TCP. The reward is defined as

$$r=-w\cdot area(abs\left(e_{x}\right))$$

where *r* represents the reward and *w* is the weight of the element. In this case, since the reward pertains to a single element, the weight is applied to scale the reward relative to the termination rewards, which are defined as

$$r_{t}=\left\{\begin{array}{cc} w_{F_{m}} & \;\mathrm{if}\;F_{m}>F_{m_{max}}\\ w_{\ddot{x}} & \;\mathrm{if}\;\ddot{x}>{\ddot{x}}_{max} \end{array}\right.$$

**Figure 5:**
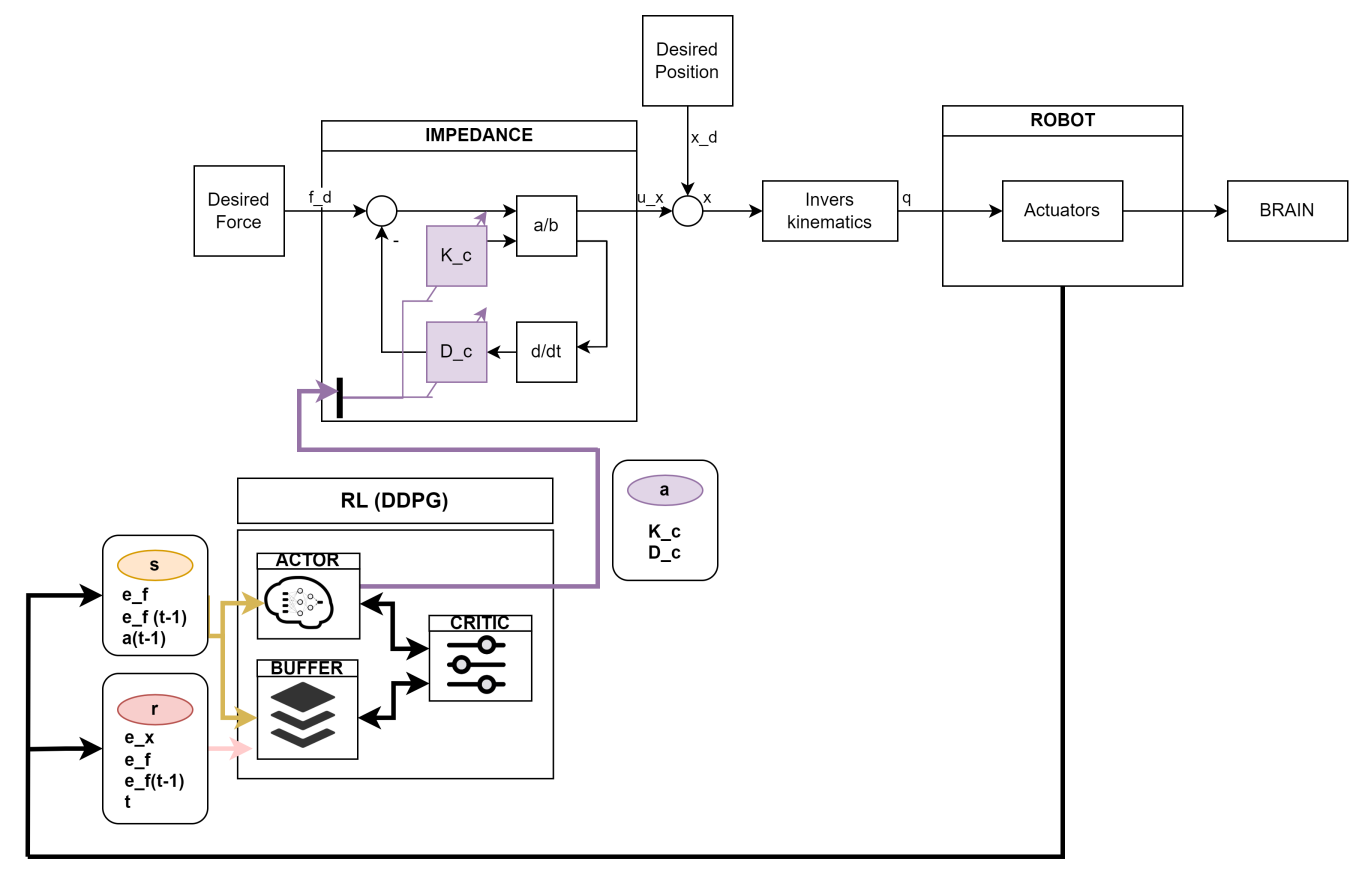
Reinforcement learning-based controller.

The resulting control system is shown in Figure 5. The RL publishes the calculated action: the stiffness and damping of the controller, as well as the desired force. The impedance controller, in turn, calculates the position command with the action of the RL. The light blue ovals represent all the variables that are published by the impedance controller for the RL to read. The purple oval and lines represent the action of the RL. The yellow line represents the state of the RL.

Despite the advantages of the original DDPG algorithm, its performance and robustness were insufficient. After a certain number of retractions, the original DDPG algorithm became unstable, causing the robot to perform abrupt movements. Even during the stable phase of the simulation, the results were unsatisfactory. Therefore, several improvements were made to the original DDPG algorithm. One key improvement involved modifying the buffer overwriting method. In the original DDPG algorithm, the buffer was overwritten sequentially, meaning that once the buffer reached its maximum capacity, new samples would replace the oldest ones in order. Better results were achieved using an RMSE-based method, specifically the normalized state-action RMSE. The purpose of this overwriting method is to prevent the buffer from being filled with similar samples, particularly those from the steady-state phase of the retraction. This method calculates the normalized RMSE between the current state and all the samples in the buffer, as well as the new current state. It then selects the samples with the smallest RMSE values and calculates the normalized RMSE between their actions and the new action. Finally, it selects the sample with the smallest action RMSE and overwrites that sample.

Regarding the configuration of the DDPG algorithm, it had a major role in the stability of the system and performance of the DDPG algorithm. After analyzing the system and the effect of the configuration’s parameters, the resulting configuration was defined as shown in Table 1:

## 4. Results

### 4.1. Simulation Results

To optimize the learning process of the system’s behavior, the control algorithm was trained using brain models with varying impedance. Specifically, the stiffness and damping of the brain model were adjusted between retractions during the simulation. As shown in Figure 2b, the system’s behavior remains quite similar for brain damping values between 0.005 $\frac{\mathrm{Ns}}{m}$ and 1 $\frac{\mathrm{Ns}}{m}$. The goal of the simulation environment is to enable the control algorithm to learn the system’s behavior effectively. To prevent the pre-training phase from becoming overly time-consuming, the brain model’s damping and stiffness were set within the ranges of 1≤x≤10 and 1≤x≤100, respectively, during pre-training. Since the robot is significantly stiffer than the brain model, the robot’s stiffness and damping were set to 50,000 $\frac{N}{m}$ and 0.1 $\frac{\mathrm{Ns}}{m}$, respectively.

**Figure 6:**
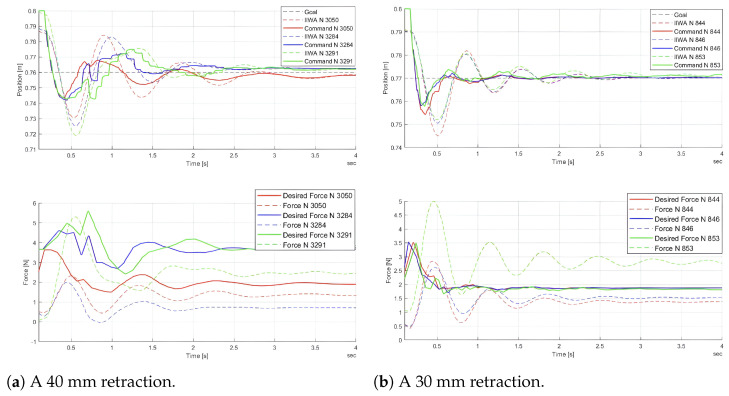
The most effective brain retractions.

The most suitable DDPG algorithm was trained through two simulations, with the objectives of retracting 40 mm and 30 mm, respectively. Figure 6 displays the most effective brain retractions from each simulation. Specifically, the first simulation includes retractions 3050, 3284, and 3291, while the second includes retractions 844, 846, and 853. The upper graphs show the goal position, command position, and the position of the robot’s TCP (tool center point). The lower graphs illustrate the desired force and the measured force at the TCP. Both the command position and desired force are computed by the control system.

The position graphs demonstrate how the control system has learned the system dynamics, effectively counteracting the inertia of both the brain and the robot to minimize overshoot. The maximum position error observed in both position graphs in Figure 6 is 8.75% and 2.667%, respectively. A force limit of 6 N was set for all simulations, and this limit was not exceeded by either the desired or measured forces shown in the graphs. In cases where the force exceeded the limit, the RL algorithm stored the state and action leading to the termination state for further learning. The control system would then reset the environment and initiate the retraction process again.

The damping value of the brain model varies between retractions, which explains why some retractions exhibit greater oscillations than others. In the real environment, the oscillation is almost negligible, indicating that the damping of the brain is minimal. However, the damping in the simulations was not adjusted, as the purpose of the simulations was to learn the behavior of the system, rather than to replicate the real environment precisely.

### 4.2. Real Results

**Figure 7:**
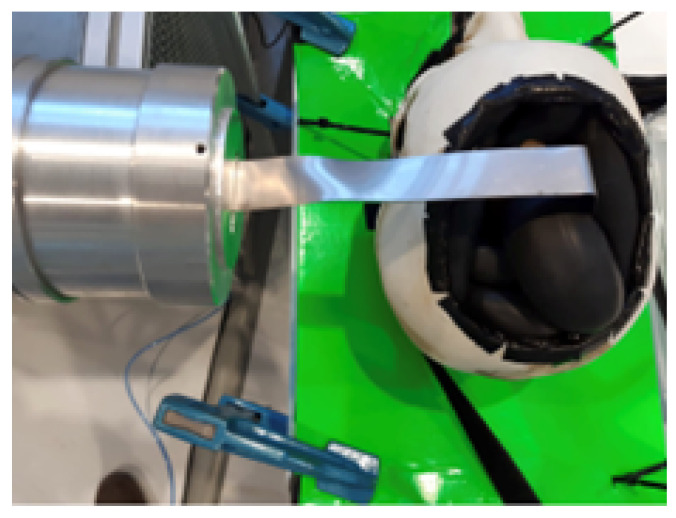
Retraction operation in the real validation environment.

The trained DDPG algorithm described in the previous section was applied to the real environment with a retraction objective of 30 mm, where the home position was set at 0.68 m on the *x*-axis, and the goal was 0.65 m. By using relative positions or position errors, the robot’s home position can be defined at any point within its workspace without compromising the performance of the DDPG algorithm. These retraction operations were carried out in a real validation environment, as shown in Figure 7. In this figure, the robot can be seen performing the retraction with the retraction tool on a brain simulated by the pneumatic system with variable characteristics.

**Figure 8:**
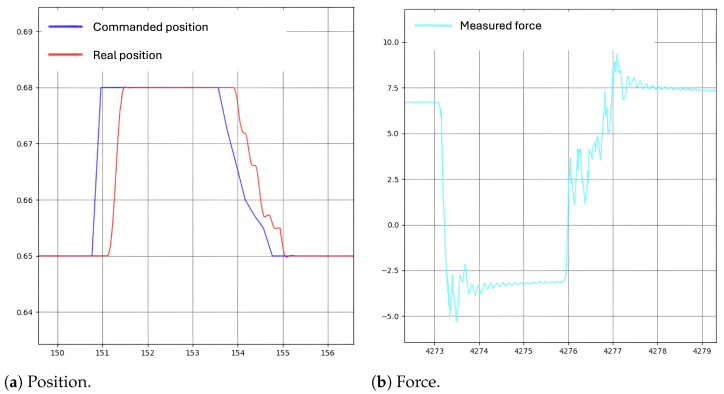
A 30 mm retraction.

The result of one of these retraction operations is shown in Figure 8). Several retraction tests representing different conditions and objectives (such as brain stiffness and retraction positions) were performed, all with similar results. As seen in Figure 8a), the controller’s command (blue) successfully directs the robot (red) to the target position. The more realistic damping of the brain in the real environment has resulted in exceptional performance from both the DDPG algorithm and the control system. Figure 8b) illustrates the forces during the retraction. The maximum tolerated force is set to 8N, and since this threshold is not exceeded, the controller continues the retraction process. If the force limit is breached, the controller halts the retraction and commands the robot to return to its home position.

## 5. Discussion

As previously mentioned, the performance and robustness of the original DDPG algorithm deteriorated after several retractions, leading to abrupt movements. One of the primary causes of these sudden movements was overfitting, a common issue in neural networks (NNs). Overfitting occurs when an NN is trained on similar data samples, causing it to replicate the behavior of those data perfectly. However, when new or different data are introduced, the NN’s output becomes unpredictable. To mitigate overfitting, the update rate was decreased, and the buffer’s overwriting method was modified to ensure that exploration samples were not overwritten. Nonetheless, placing too much emphasis on exploration could prevent the actor and critic from adequately responding to the most common states during retractions. Therefore, the balance between exploration and exploitation was analyzed and improved. Another modification aimed at preventing abrupt movements was the adjustment of the reward function. Initially, the reward was designed to penalize oscillations using the variables $\dot{x}$ (velocity) and ${\dot{F}}_{m}$ (rate of change in force). However, $\dot{x}$ was removed from the reward. Similarly, ${\dot{F}}_{m}$ was removed for the same reasons. Additionally, both force and position were initially included in the reward function, but since force and position are proportional, including both was redundant and made it more difficult for the critic to learn the reward function effectively, so force ${\dot{F}}_{m}$ was also removed.

Furthermore, specific elements of the DDPG algorithm were modified to address underperformance in the robot. Initially, the desired force was defined as a constant, meaning that the RL had to calculate the controller’s impedance values while accounting for the limitations of the desired force. This approach left only two parametrizable variables in the impedance equation, which hindered learning and negatively impacted the DDPG’s performance. To resolve this, the desired force was added as a parametrizable variable in the action space.

Overall, the results obtained in the real environment demonstrate the ability of the proposed control algorithm to correctly perform retraction operations under multiple retraction conditions based on its prior knowledge obtained in the learning phase.

## 6. Conclusions

This paper presents a DDPG-based adaptive impedance controller developed for the control of a KUKA LBR iiwa 14 collaborative robot, specifically designed for brain retraction procedures. The controller was developed, refined, and trained within a simulation environment that modeled the forces generated during contact between the robot and the brain and calculated contact positions. After completing the training in the simulation environment, the DDPG algorithm was deployed in a real environment.

The DDPG algorithm, equipped with a buffer overwriting method based on the normalized RMSE, was trained against a contact model and is capable of performing retractions to the defined objective, particularly in the real environment. By simulating a wide range of brain damping values, the control system effectively learned the system’s behavior, resulting in minimal oscillation when applied to the real environment with realistic brain damping. On the other hand, the experimentation in a real setting also revealed that the simplified consideration of a single point of contact to guide the learning process is adequate for successful retraction operations.

Moreover, the retractions were successfully executed even when the desired force was unknown, as it could be dynamically calculated by the RL algorithm. In other words, the same position could be achieved with different desired force values by adjusting the impedance of the controller, making it less critical to minimize the force error.

The configured DDPG improves both the stability and robustness of the base algorithm, allowing it to perform multiple retractions without causing harm to the patient.

Additionally, the algorithm was implemented and validated on a more cost-effective alternative to specialized medical or surgical robots.

## Figures and Tables

**Table 1:** DDPG hyperparameters.

Variable	Value
Buffer size	20,000
Update every	500
Exploration phase steps	5000
Update batch size	100
Actor NN size	4 × 60
Critic NN size	4 × 66
Actor learning rate	1 × 10^−4^
Critic learning rate	0.01
*w*	10,000
$w_{F_{m}}$	−5000
$F_{m_{max}}$	8
$w_{\ddot{x}}$	−2000
${\ddot{x}}_{max}$	500

## Data Availability

Data are contained within the article.
